# Plasma membrane protein trafficking in plant–microbe interactions: a plant cell point of view

**DOI:** 10.3389/fpls.2014.00735

**Published:** 2014-12-22

**Authors:** Nathalie Leborgne-Castel, Karim Bouhidel

**Affiliations:** UMR1347 Agroécologie AgroSup/INRA/uB, ERL CNRS 6300, Université de Bourgogne, Dijon, France

**Keywords:** plasma membrane, vesicular trafficking, plant–microbe interactions, signaling proteins, sugar transporters

## Abstract

In order to ensure their physiological and cellular functions, plasma membrane (PM) proteins must be properly conveyed from their site of synthesis, i.e., the endoplasmic reticulum, to their final destination, the PM, through the secretory pathway. PM protein homeostasis also relies on recycling and/or degradation, two processes that are initiated by endocytosis. Vesicular membrane trafficking events to and from the PM have been shown to be altered when plant cells are exposed to mutualistic or pathogenic microbes. In this review, we will describe the fine-tune regulation of such alterations, and their consequence in PM protein activity. We will consider the formation of intracellular perimicrobial compartments, the PM protein trafficking machinery of the host, and the delivery or retrieval of signaling and transport proteins such as pattern-recognition receptors, producers of reactive oxygen species, and sugar transporters.

## INTRODUCTION

Plants co-exist with a vast diversity of mutualistic or pathogenic microbes such as bacteria, fungi, and oomycetes. Most of the land plants form a mutualistic relationship with symbiotic fungi such as mycorrhizal fungi, and legumes with rhizobia bacteria. Only a small fraction of plant–microbe encounters results in pathogenic relationship.

Indeed, the plants have developed intricate defense mechanisms, referred to as innate immunity, to identify and defend themselves against a wide range of pathogens (Dodds and Rathjen, 2010). The plasma membrane (PM) of plant cells possesses pathogen-recognition receptors (PRR) that recognize pathogen/microbe-associated molecular patterns (P/MAMP), formerly called elicitors, and initiate a pathogen-associated immunity (PTI; Boller and Felix, 2009). PTI, as a basal resistance, limits the invasion and propagation of pathogens. Adapted pathogens are able to suppress PTI by secreting virulence effectors that remain in the apoplast or penetrate the host cell cytoplasm, favoring pathogen survival, and mediating effector-triggered susceptibility. In turn, to confine and eliminate adapted pathogens, plants have evolved resistance (R) proteins that directly or indirectly recognize effectors and thwart pathogen attack. This second line of defense is named effector-triggered immunity (ETI) or R gene-mediated resistance (Win et al., 2012).

Many plant defense responses to infection are shared by PTI and ETI resistance such as protein phosphorylation, changes in ion fluxes, production of reactive oxygen species (ROS), synthesis of antimicrobial compounds, and pathogenesis-related proteins associated with cell wall reinforcement. ETI elicits more prolonged and robust immune responses than PTI, and is more often associated with the hypersensitive cell death that inhibits pathogen growth (Tsuda and Katagiri, 2010).

Accordingly, the plant cell PM contains integral or peripheral proteins that regulate plant biotic interactions by recognizing microbes, activating subsequent signaling cascades, and controlling cellular entrance and exit of molecules.

To reach the PM, proteins travel along the endomembrane system. They are first synthesized in the endoplasmic reticulum (ER) and then transported along the secretory pathway through the Golgi apparatus and the *trans*-Golgi network (TGN) to be delivered to the PM by exocytosis (Peer, 2011). Once at the PM, proteins remain there or are taken up by endocytosis, and either targeted to the lytic vacuole for degradation, or stored in endocytic compartments and recycled back to the cell surface when needed. The main and best-studied endocytic pathway in plant cells is the clathrin-dependent pathway (Chen et al., 2011). A membrane microdomain-associated endocytic pathway has also been described in plant that may involve specific microdomain proteins such as flotillins (Li et al., 2012).

Protein trafficking through the secretory and endocytic pathways relies on membrane-bound vesicles and a complex set of proteins involved in vesicle formation, transport, docking, and fusion with the respective target membrane (Bonifacino and Glick, 2004). By adjusting vesicle trafficking, plant cells can quickly respond to microbe confrontation. This topic has aroused considerable interest in recent years as shown by numerous reviews (Robatzek, 2007; Ivanov et al., 2010; Leborgne-Castel et al., 2010; Dörmann et al., 2014; Inada and Ueda, 2014; Teh and Hofius, 2014). Here, we review recent progress in our understanding of how both mutualistic and hostile plant–microbe encounters modulate membrane trafficking pathways to reshape the host PM. A special emphasis is placed on membrane trafficking regulators, and on integral PM proteins implicated in cell signaling and nutrient transport.

## FORMATION OF PERIMICROBIAL COMPARTMENTS

**FIGURE 1:**
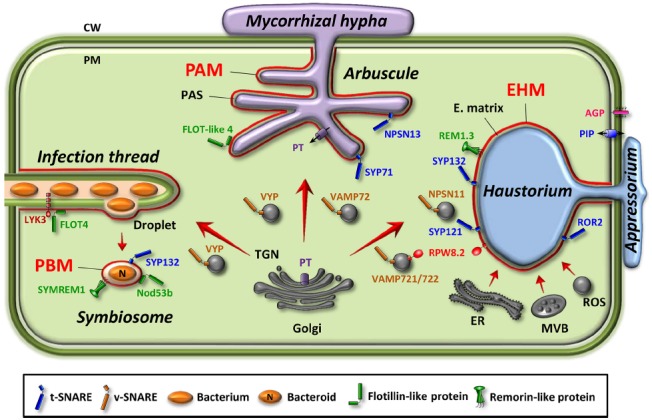
**Perimicrobial membranes in three types of plant–microbe interactions.** Location of proteins specifically targeted to or excluded from the perimicrobial membranes are indicated. Endomembrane compartments or vesicles involved are illustrated. Small GTPases and exocyst subunits are not represented. AGP, arabinogalactan protein; CW, cell wall; EHM, extrahaustorial membrane; E. matrix, extrahaustorial matrix; ER, endoplasmic reticulum; FLOT, flotillin; LYK3, lysM-family receptor-like kinase from *Medicago*; MVB, multivesicular body; N, fixed-nitrogen; Nod53b, nodule-specific 53-kDa protein from soybean; NPSN, novel plant SNARE, PAM, periarbuscular membrane; PAS, periarbuscular space; PBM, peribacteroid membrane; PIP, plasma membrane intrinsic protein (aquaporin); PM, plasma membrane; PT, phosphate transporter; REM, remorin; ROR2, required for mlo-specified resistance2 syntaxin; ROS, reactive oxygen species; RPW8.2, resistance powdery mildew8.2; SYMREM, symbiotic remorin1; SYP, syntaxin of plant; TGN, *trans*-Golgi network; VAMP, vesicle-associated membrane protein; VYP, vapyrin.

Plants can establish intracellular associations with mutualistic as well as pathogenic microbes. The intracellular accommodation of microbes allows them to gain access to host nutrients. In a mutualistic relationship, the host cell also benefits from specific metabolites originating from the microbes. Microbes penetrate the cell wall and differentiate specialized intracellular feeding compartments by invagination and extension of the host cell PM to colonize the intracellular space (Figure 1). The perimicrobial compartment refers to the membrane of the host cell at the interface between the host cytoplasm and the microbe. Perimicrobial compartments are highly diverse in shape but their biogenesis relies on similar mechanisms, mainly an intricate balance between endocytosis and exocytosis (Ivanov et al., 2010).

### PERIBACTEROID MEMBRANE

Mutualistic association of legume plants with rhizobia culminates in the organogenesis of nitrogen-fixing root nodules. Rhizobia are first hosted in an intracellular infection thread (IT) that guides them through the root cell layers. The developing IT results from the local hydrolysis of the plant cell walls and invagination of the PM (Gage, 2004). When the IT reaches the nodule primordium, rhizobia are released from unwalled IT droplets into the plant cell cytoplasm by an endocytosis-like process to form the symbiosome (Figure 1). The symbiosome is an organelle-like compartment surrounded by the peribacteroid membrane (PBM) where rhizobia convert atmospheric nitrogen into ammonia. Numerous symbiosomes occupy each nodule cell; therefore an enormous amount of membrane material must be delivered to the PBM for symbiosome development and maintenance. Vesicle trafficking delivers new lipid and protein materials to the PBM, thus imparting a distinct biochemical identity to this membrane (Ivanov et al., 2010, 2012). In particular, remorin or flotillin proteins, two classes of membrane microdomain-associated proteins, localize to the PBM and are necessary for nodule organization and function (Haney and Long, 2010; Lefebvre et al., 2010; Haney et al., 2011). The actual function of these two classes of proteins remains unclear but they may play a role in IT initiation and elongation, and the entry of rhizobia.

### PERIARBUSCULAR MEMBRANE

Mycorrhizal fungi form arbuscular mycorrhiza (AM) associated with plant roots. Intracellular fungal hyphae are enveloped by the periarbuscular membrane (PAM; Figure 1) that corresponds to an ∼10-fold expansion of the host cell PM (Alexander et al., 1989; Bonfante and Genre, 2010). The mechanisms leading to PAM biogenesis have been investigated, showing that pre-penetration responses and symbiotic interface construction are associated with extensive membrane dynamics as clathrin (Genre and Bonfante, 1999) and exocytosis markers accumulate at the site of PAM biogenesis (Genre et al., 2012). Moreover, gene expression reprogramming during AM symbiosis concerns, in addition to nutrient transporters, proteins involved in membrane dynamics, cell wall synthesis, and protein turnover (Guether et al., 2009; Harrison, 2012; Lota et al., 2013), thus suggesting an intense trafficking of cellular membrane components and cell wall precursors to the extending PAM. Recently, a qualitative proteome analysis of the *Medicago* root upon AM symbiosis evidenced flotillin-like proteins and proteins involved in vesicle trafficking supporting the importance of membrane trafficking events in mycorrhiza establishment and functioning (Abdallah et al., 2014). Although the PAM is continuous with the peripheral host PM, asymmetric distribution of transport proteins has been demonstrated. The prototypal protein of such asymmetric distribution is the phosphate transporter MtPT4 from *Medicago* that is found only in PAM without trace in the peripheral host PM in developed arbuscules (Harrison et al., 2002). Same focalized localization was observed for OsPT11 in rice (Kobae and Hata, 2010). MtPT4 polar localization results from a transient reorientation of secretion via the ER and TGN compartments (Pumplin et al., 2012).

### EXTRAHAUSTORIAL MEMBRANE

During fungi and oomycete pathogen attack, the first responses of the plant cell are cell wall defensive appositions, called papillae, beneath the attempted pathogen penetration site deposited through the action of the secretory pathway and the cytoskeleton reorganization (Hückelhoven et al., 1999). Defense against penetrating pathogens also involves post-Golgi vesicles and multivesicular bodies (MVB)/late endosome for secretion of antimicrobial molecules at the host cell wall penetration site (An et al., 2006).

Adapted pathogens have evolved the ability to breach the host cell wall leading to the development of an infection structure, underneath the appressorium, from which a narrow hyphal strand grows and penetrates the plant cell wall (Koh et al., 2005). The penetration hypha then expands into the host cell cytoplasm and differentiates into a haustorium that allows the pathogen to extract nutrients and secretes effectors to suppress plant defense mechanisms (Figure 1). The haustorium is encased by a specialized membrane, the extrahaustorial membrane (EHM). Haustorium development is associated with extensive dynamics at the EHM and intense exocytosis of proteins and polysaccharides reaching the extrahaustorial matrix via the plant secretory pathway (Mendgen et al., 1995; Meyer et al., 2009). In addition, transmission electron microscopy has revealed swollen tubules and cisternae of plant ER in a very close proximity to EHM (Koh et al., 2005; Micali et al., 2011) that could allow a direct transfer of membrane components to EHM without involvement of the Golgi apparatus (Leckie et al., 1995; Figure 1). EHM is believed to originate from the remodeling of the host cell PM but an alternative hypothesis proposes that EHM is formed via *de novo* biogenesis (Koh et al., 2005). Many plant PM resident proteins, such as arabinogalactan protein (Micali et al., 2011) or aquaporins, are excluded from the EHM of fungi (Koh et al., 2005; O’Connell and Panstruga, 2006) and oomycetes (Caillaud et al., 2012; Lu et al., 2012). Some proteins, however, are specifically targeted to the mature EHM such as the resistance to powdery mildew8.2 (RPW8.2) in *Arabidopsis thaliana* (Micali et al., 2011; Wang et al., 2013), and the REM1.3 remorin in *Nicotiana benthamiana* that promotes susceptibility to *Phytophthora infestans* (Bozkurt et al., 2014) (Figure 1).

### OPEN QUESTIONS

Differentiation of a perimicrobial compartment seems to rely heavily on polarized secretion (Ivanov et al., 2012; Pumplin et al., 2012). Is there an identity checkpoint along the secretory pathway that involves specific chaperones to retain some proteins and let go others such as the periarbuscular phosphate transporters MtPT4 and OsPT11, or the EHM RWP8.2 protein? Is polarized secretion also controlled at the target membrane? What is the role of the scaffolding proteins flotillin and remorin that are known to be associated with sterol- and sphingolipid-enriched microdomains? As focal exocytosis in animal cells depends upon the accumulation of *N*-ethylmaleimide-sensitive factor attachment protein receptors (SNAREs; see below) in PM microdomains (Puri and Roche, 2006; Tsai et al., 2007), it is tempting to speculate that, in plants, a similar phenomenon control the docking and fusion of vesicles loaded with perimicrobial membrane-specific proteins.

Another challenge will be to determine to what extent effectors secreted from mutualistic and pathogenic microbes manipulate the host secretory pathway and reorient secretion toward the perimicrobial membrane. The fact that the oomycete effector AVRblb2 colocalizes with REM1.3 in perihaustorial domains (Bozkurt et al., 2014) suggests that pathogens exploit the lateral organization of the host membrane to accommodate infection structures.

## REGULATORS OF VESICLE TRAFFICKING

The transport of membrane components from one compartment to another within the endomembrane system is achieved through the continuous budding, movement and fusion of vesicles. The specificity of vesicular transport is in part ensured by small GTPases of the Rab, ARF (ADP-ribosylation factor; Nielsen et al., 2008), and ROP (Rho of plants)/RAC (Ras-related C3 botulinum toxin substrate; Yalovsky et al., 2008) subfamilies that cycle between an active GTP-bound state associated with membranes and an inactive GDP-bound state present in the cytosol (Rutherford and Moore, 2002). Rab and ARF GTPases work with soluble SNARE proteins to promote membrane fusion (Uemura et al., 2004; Bassham and Blatt, 2008). It is the pairing of complementary SNAREs associated with the vesicle and the target membrane that overcomes the repulsive forces between the two lipid bilayers and drives their fusion. In addition to small GTPases and SNAREs, vesicle tethering factors are necessary for the initial contact and subsequent fusion of the transport vesicle to its target membrane (Yu and Hughson, 2010). One of them is the exocyst, a protein complex composed of eight subunits (SEC3, SEC5, SEC6, SEC8, SEC10, SEC15, EXO70, and EXO84) in *Arabidopsis* (Zárský et al., 2013) that mediates the docking of exocytic vesicles to the PM in relation with the cytoskeleton (Synek et al., 2014) before SNARE complex formation (Fendrych et al., 2013).

As developed below, all three types of intracellular trafficking regulators have been found to be implicated in plant–microbe interactions.

### SMALL GTPases

From the early stages of plant–rhizobial interaction, a major reprogramming of plant genes associated with the initiation of vesicle trafficking is induced that includes Rab and ARF genes and leads to IT development (Peleg-Grossman et al., 2007).

Rab GTPases that are involved in post-Golgi secretion to the PM appear to play a role in plant defense (Speth et al., 2009) and to regulate autophagy and immunity-associated hypersensitive response (Kwon et al., 2013). ARF GTPases have been implicated in preinvasive penetration resistance against powdery mildew in barley (Böhlenius et al., 2010).

ROP/RAC GTPases that control polarized cell growth have been implicated in directing exocytic vesicles via the cytoskeleton to sites of fast membrane expansion during interaction of fungal pathogens with barley (Opalski et al., 2005; Pathuri et al., 2008), *Arabidopsis* (Poraty-Gavra et al., 2013), and rice (Chen et al., 2010a), or mycorrhizal and oomycete root colonization in barley (Kiirika et al., 2012). A role for ROP GTPases in defense-related endocytic processes can also be suspected from the demonstration of the involvement of clathrin-mediated endocytosis in plant defense (Leborgne-Castel et al., 2010), and ROP GTPases regulation of clathrin-mediated endocytosis in *Arabidopsis* roots (Chen et al., 2012).

Small GTPases are activated and deactivated through interaction with guanine nucleotide exchange factors (GEFs) that catalyze the exchange of bound GDP with cytosolic GTP, and GTPase activating proteins (GAPs) that enhance GTP hydrolysis (Cherfils and Zeghouf, 2013).

Inhibition of host vesicle-mediated trafficking by targeting activators of GTPase proteins is one strategy of microbes to hinder plant defenses. Indeed, one well-known fungal toxin is brefeldin A, a potent inhibitor of ER-to-Golgi protein transport that targets ARF-GEF proteins (Anders and Jürgens, 2008). The bacterial effector HopM1 from *Pseudomonas syringae* destabilizes the *Arabidopsis* ARF-GEF AtMIN7 through the host 26S proteasome that leads to suppression of immunity (Tanaka et al., 2009; Nomura et al., 2011). Interestingly, both the effector and its target have been localized in the TGN suggesting a role for this compartment in defense. A candidate effector from *Blumeria graminis* was identified as an interactor of a barley ARF-GAP, suggesting interference with defense-associated host vesicle trafficking. A mutation in the orthologous gene in *Arabidopsis* allows higher entry of powdery mildew agent, *Erysiphe pisi*, thus confirming that this protein is required for defense against non-adapted fungal pathogens (Schmidt et al., 2014). Interestingly, the same mutant exhibits elevated resistance to the adapted downy mildew pathogen *Hyaloperonospora arabidopsidis*, thus signifying that a single ARF-GAP may function antagonistically in defense against adapted and non-adapted pathogens.

### SNARE PROTEINS

PTI-dependent resistance against pathogenic fungi is characterized by the secretion of antimicrobial components into the plant apoplast, a process that requires the activity of several SNARE proteins. SYP121/PEN1 (for PENETRATION), which is the most studied PM-resident SNARE, plays a pivotal role in cell wall-based resistance against the powdery mildew agent, *Blumeria graminis*, in *Arabidopsis* (Collins et al., 2003; Assaad et al., 2004; Zhang et al., 2007; Pajonk et al., 2008), barley (Bhat et al., 2005), and grapevine (Feechan et al., 2013). The potato homolog of SYP121, StSYR1, also participates in the formation of callose-containing papillae for secretory defense responses of potato against the oomycete *Phytophthora infestans* (Eschen-Lippold et al., 2012). A loss-of-function mutation in the SYP121/PEN1 gene was recently shown to decrease hypersensitive cell death triggered by oomycete and bacterial effectors (Johansson et al., 2014) suggesting that this SNARE is also involved in ETI response.

SYP121/PEN1 forms a ternary SNARE complex with the adaptor protein SNAP33 and the sequence-related vesicle-associated membrane proteins VAMP721/722 at the PM that is necessary for pre-invasive immune responses in barley and *Arabidopsis* (Kwon et al., 2008). The R-protein RPW8.2, which is specifically targeted to the EHM, is transiently located on VAMP721/722 vesicles before incorporation into the EHM of mature haustoria (Figure 1). This reveals that VAMP721/722 vesicles are not only engaged in pre-invasive defense at the cell periphery but also in post-invasive defense to carry the resistance protein RPW8.2 at the EHM (Kim et al., 2014).

Moreover, the PM SNARE SYP132 has been identified to contribute to defense against bacterial pathogens in *N. benthamiana*, notably by participating in the secretion of antimicrobial proteins to fight the extracellular infection (Kalde et al., 2007). SYP132 also interacts with VAMP721/722 in the constitutive secretion pathway during *Arabidopsis* growth. In untreated plants, VAMP721/722 barely accumulate at the PM as they move continuously to and from the PM, and are rapidly degraded by the 26S proteasome (Yun et al., 2013). Upon application of flg22, the bacterium-derived active peptide of flagellin, which induces immune response as well as growth inhibition in plants, VAMP721/722 appears to be protected from proteosomal degradation (Yun et al., 2013). This regulation suggests that growth inhibition during MAMP responses is induced by the reorganization of the secretory pathway for defense. This could open new perspectives to reduce defense-associated fitness costs in crop plants (Sup Yun et al., 2013).

TaNPSN11, a wheat SNARE belonging to the plant-specific novel plant SNARE (NPSN) group (Sanderfoot et al., 2000), has been recently associated with defense against pathogens (Wang et al., 2014). TaNPSN11 is present in vesicular structures near cell membranes (Figure 1) in contact with fungal hyphae when wheat leaves are challenged with the stripe rust agent *Puccinia striiformis* f. sp. *tritici* (Wang et al., 2014). TaNPSN11 interacts with TaSYP132 in the TGN and PM, and when downregulated, confers a reduced resistance to an avirulent race of the fungal pathogen. TaNPSN11 thus appears to be involved in vesicle-mediated resistance to stripe rust. NPSN11 was initially described as a component of the fusion machinery involved in cell plate formation in *Arabidopsis* (Zheng et al., 2002; El Kasmi et al., 2013). The work of Wang et al. (2014) suggests that this plant specific SNARE may have a dual role during cytokinesis and plant-pathogen interactions.

Finally, a recent interactome analysis of SNAREs, using *A. thaliana* plants expressing GFP-tagged SYP/SNAREs, has identified several disease resistance proteins of the TIR-NBS class as potential interactants (Fujiwara et al., 2014), a result that argues for a role for SNAREs in ETI.

The involvement of PM SNARE proteins is also established for plant-mutualist interactions. Numerous membrane proteins are proposed to be targeted to the symbiosome membrane by SNAREs via the Golgi apparatus (Catalano et al., 2004). Notably, MtSYP132 from *Medicago* is localized to the IT and infection droplet membranes indicating that this SNARE might have a dual role in IT growth and symbiosome formation (Figure 1; Catalano et al., 2007). In *Lotus japonicus*, SYP71, which is expressed in vascular tissue, has been shown to play a pivotal role in symbiotic nitrogen fixation in nodules as *Ljsyp71* mutant presents nodulation defects (Hakoyama et al., 2012).

Pre-penetration apparatus formation during AM infection is less documented but probably involves the main components of the exocytic machinery, with a major participation of the Golgi apparatus, such as proteins of the VAMP72 family as revealed by both transmission electron microscopy and *in vivo* GFP imaging (Figure 1; Genre et al., 2012). This data is supported by the recent identification of target-SNAREs (NSPNS13 and SYP71) and vesicle-SNAREs from VAMP72 protein family that is required for arbuscule development (Ivanov et al., 2012; Abdallah et al., 2014). Moreover, a vesicle-associated membrane protein, vapyrin, which contains several ankyrin protein–protein interaction repeat motifs, was shown to be involved in cellular remodeling during AM symbiosis (Pumplin et al., 2010), and also appears to be essential for the rhizobial infection in *Medicago truncatula* (Murray et al., 2011; Figure 1). The role of vapyrin in membrane trafficking is still questioned. The observation that vapyrin-GFP localizes to mobile puncta in mycorrhizal roots suggests that vapyrin may be associated with vesicles in exocytosis-driven polar tip growth in response to external signals such as Nod or Myc factors (Feddermann et al., 2010; Pumplin et al., 2010).

### EXOCYST COMPLEX

Several recent findings accredit the role of the exocyst complex as a new regulator of plant–microbe interactions. Expression of genes encoding exocyst subunits, including EXO70B2, H1, H2, and H7, is highly responsive to different pathogens or elicitor treatment such as flg22 and elf18, the active peptide of the prokaryotic elongation factor EF-Tu, in *Arabidopsis* (Pečenková et al., 2011). Likewise, cryptogein, a defense elicitor from the oomycete *Phytophthora cryptogea*, triggers a 10-fold up-regulation of *EXOH4* in tobacco cells (Bouhidel, unpublished data). Moreover, accumulation of the exocyst subunit EXO84b was revealed at the site of PAM biogenesis in AM (Genre et al., 2012).

Interestingly, *Arabidopsis exo70B2* and *exo70H1* mutant lines display a higher susceptibility to *Pseudomonas syringae* pv. *maculicola* infection, and *exo70B2* mutants present an increase in the proportion of abnormal papillae after infection with *Blumeria graminis* f. sp *hordei* (Pečenková et al., 2011). Exo70B2 is also identified as a target of the plant U-box-type ubiquitin ligase 22 (PUB22), which is a negative regulator of PAMP-triggered responses, and it is supposed that PUB22-mediated ubiquitination and degradation of Exo70B2 contributes to the attenuation of PAMP-induced signaling (Stegmann et al., 2012).

In addition, RIN4, a well-known regulator of bacterial-triggered ETI as a guardee interacting with multiple plant R-proteins, has been shown to interact with components of the exocyst complex, raising the hypothesis that RIN4 is involved in defense-associated vesicle trafficking (Afzal et al., 2013).

### OPEN QUESTIONS

Compelling progress has been made in recent years in our understanding of the role of membrane trafficking in plant–microbe interactions but we still do have a fragmentary picture of the processes and molecular actors that are involved in the regulation of vesicle trafficking. One should ask whether there is some unity in the mechanisms of vesicular transport. Some studies have already helped to put together some pieces of the puzzle. v-SNAREs of the VAMP72 family have been identified as common symbiotic regulators in exocytotic vesicle trafficking (Ivanov et al., 2012) and also appear to be involved in the exocytotic pathway that leads to EHM biogenesis (Kwon et al., 2008). A ternary SNARE complex between SYP121/PEN1, SNAP33, and VAMP721/722 at the PM has been identified that mediates the pre-invasion resistance against non-adapted pathogens (Kwon et al., 2008). Is there a defense- or a symbiosis-related “trafficking code,” similar to the well-known genetic and histone codes, that associates SNAREs, exocyst subunits and small GTPases to guide the intracellular journey of exocytic and endocytic vesicles during plant–microbe interactions? The identification of interactants of already known regulators may pave the way for a better understanding of the molecular mechanisms that control such membrane trafficking events.

## TRAFFICKING OF INTEGRAL PM PROTEINS

Now that small GTPases, SNAREs, and exocyst subunits are established as important trafficking regulators in the plant cell responses to microbes, it is important to have an overview of the subcellular trafficking of mutualism- and defense-associated PM proteins. We describe hereafter selected examples of spatial regulation involving major actors in the responses of plants to microbes such as PRRs, respiratory burst oxygen species homologs (RBOHs), and sugar transporters.

### PATTERN RECOGNITION RECEPTORS

The plant cell PM contains a plethora of receptors (PRRs) that sense changes in the biotic environment and trigger specific signaling cascades and physiological outputs inside the cell. PRRs do this by recognizing evolutionarily conserved MAMPs/elicitors or molecules released from damaged plant cells. PRRs are divided into two classes: leucine-rich repeat receptor-like proteins (LRR-RLPs) and receptor-like kinases (LRR-RLKs). Increasing evidence supports the claim that PRRs are part of multi-protein complexes associating regulatory proteins (Monaghan and Zipfel, 2012).

#### Control of ER exit

The ER is the first compartment of the secretory pathway. It ensures the quality-control process (ER-QC) for folding and maturation of client secretory proteins during and after their biosynthesis (Vitale and Boston, 2008). Many ER proteins such as chaperones involved in protein folding or components of the *N*-glycosylation pathway, which permit a correct trimming of oligosaccharides on nascent proteins, are major actors in the ER-quality control.

ER-QC dysfunction leads to folding defect of PRRs that affect their quality, accumulation, and/or complex formation at the PM necessary for signaling competency, and consequently for plant resistance to pathogens. The prototypal PRR that has been shown to require such control is EFR, the receptor of EF-Tu in *Arabidopsis*. A recent review suggests that, owing to the diversity of ER-proteins engaged, the anterograde trafficking of PRR may be a potential point of regulation to generate appropriate defense responses (Tintor and Saijo, 2014).

During symbiosis in *M. truncatula*, the trafficking of the lysin motif receptor-like kinase NFP, which is required for perception of Rhizobium Nod factors and establishment of symbiosis, is highly sensitive to regulation in the ER. Notably, NFP biological function depends upon the formation of disulfide bridges mediated by the ER protein disulfide isomerases in the receptor extracellular region (Lefebvre et al., 2012).

In addition to the ER-QC components, ER proteins of the reticulon-like protein family (RTNBs), which take part of ER network structuration (Nziengui and Schoefs, 2009), have been shown to interact with the FLAGELIN SENSING 2 receptor (FLS2; Lee et al., 2011). RTNB mutations affect both FLS2 and EFR signaling (Lee et al., 2011; Popescu, 2012) suggesting a role for RTNBs in PRR exit from the ER and trafficking along the secretory pathway.

Recently, ACD6 (ACCELERATED CELL DEATH6), a multipass membrane protein with an ankyrin domain, has been involved in the formation of a complex with FLS2 at the ER to promote FLS2 targeting to the PM and immune response under the signaling defense hormone salicylic acid SA (Zhang et al., 2014).

#### Endocytosis

Environmental changes could alter the abundance of plant PM proteins by the process of endocytosis (Leborgne-Castel and Luu, 2009). Once removed from the PM, integral proteins are either targeted to the vacuole for degradation or recycled back to the PM. Ubiquitination is used as a sorting signal that directs membrane proteins for degradation. Once internalized, ubiquitinated proteins are delivered via the TGN/early endosome to the MVB/late endosome, where they are recognized by the endosomal sorting complex (ESCRT), and sorted into internal vesicles that are subsequently discharged into the lytic vacuole when the MVB/late endosome fuses with the tonoplast.

Internalization of cell surface receptors, via the clathrin-mediated pathway or any other endocytic pathway, would serve to terminate signaling through degradation, sustain signaling through recycling, or relay signaling inside the cell through the formation of signaling endosomes (Geldner and Robatzek, 2008).

Evidence of MAMP-inducing PRR endocytosis comes from localization studies with the flagellin receptor FLS2 (Robatzek et al., 2006). Following flg22 elicitation, GFP-tagged FLS2 is targeted to a compartment with properties intermediate between the TGN/early endosome and the MVB/late endosome in *Arabidopsis* (Beck et al., 2012) and *N. benthamiana* (Choi et al., 2013). Detailed analysis of FLS2 location revealed its presence in the MVB lumen in association with components of the ESCRT-I complex, VPS37-1, and VPS28-2 (Spallek et al., 2013). Interestingly, VPS37-1 and VPS28-2 are required for flg22-induced stomatal closure, but not for a range of other flg22-induced defense responses (i.e., ROS production, kinase activation or callose deposition), linking late endocytic trafficking of FLS2 specifically with defense-associated stomatal closure. These data are consistent with the notion that FLS2 activates separate signaling branches from its targeting to various endomembrane compartments.

Moreover, while FLS2 constitutively recycles in a brefeldin A-sensitive manner, flg22-activated receptors traffic via endosomes insensitive to brefeldin A (Beck et al., 2012). This suggests the existence of two distinct endocytic trafficking routes that depend upon FLS2 activation status and involve distinct subgroups of RabA/11 GTPases (Beck et al., 2012; Choi et al., 2013). In addition, the traffic of *de novo*-synthesized FLS2 to the PM also involves a distinct RabA/11 subgroup demonstrating that a complex regulatory system exists to properly locate FLS2 (Choi et al., 2013).

Salicylic acid treatment up-regulates the expression of clathrin proteins in maize and *Arabidopsis* (Pajerowska-Mukhtar et al., 2012; Zeng et al., 2013). Knowing the role of SA in plant immunity, it is tempting to speculate that the expression of these coat proteins is a potential component of plant immunity. Indeed, the clathrin endocytic pathway is activated by the *Phytophthora cryptogea* elicitor cryptogein in tobacco (Leborgne-Castel et al., 2008) and the *Trichoderma viride* ethylene-inducing xylanase (EIX) in tomato (Bar and Avni, 2009). In addition, activation of endocytosis may contribute to downstream elicitor signaling (Ron and Avni, 2004; Sharfman et al., 2011; Adam et al., 2012). The EIX receptor LeEIX contains a clathrin endocytic motif, that is also found in some cell surface receptors from fungi (Kawchuk et al., 2001; Fritz-Laylin et al., 2005) or bacteria (Zipfel et al., 2006). By contrast, FLS2 does not contain such motif, but harbors ubiquitination motifs that serve as signals for endocytosis and sorting to the MVBs (Robatzek et al., 2006). The flagellin-triggered endocytosis of FLS2 is altered but not blocked by tyrphostin A23 an inhibitor of the clathrin-mediated endocytosis pathway (Spallek et al., 2013), and is insensitive to SA, which inhibits clathrin-mediated endocytosis of other PM proteins (Du et al., 2013). Therefore, the endocytic pathway that target FLS2 remains to be identified.

### RESPIRATORY BURST OXIDASE HOMOLOGS

A rapid and transient production of ROS, the so-called “oxidative burst,” is a hallmark of successful recognition of plant pathogens (Lamb and Dixon, 1997). ROS are known to have antimicrobial activity, to drive cell wall reinforcement, and to act as second messengers in defense-related signaling pathways (Levine et al., 1994). Early ROS production is predominantly apoplastic and notably dependent upon PM-resident NADPH oxidases, also called RBOHs. Plants synthesize several RBOH isoforms with isoform D playing the most prevalent role during biotic stress (Torres et al., 1998; Marino et al., 2011). Owing to their pathosystem-dependent effects on hypersensitive cell death and disease resistance, RBOH-mediated ROS are now conceptualized as mediating agents in signaling pathways with opposite effects on plant defense reactions (Torres et al., 2005; Torres, 2010; Marino et al., 2011). In addition to their role in plant defense, RBOHs have also been described as a major source of ROS required for the establishment of rhizobial root nodules and mycorrhizal symbiosis (Montiel et al., 2012; Puppo et al., 2013; Arthikala et al., 2014).

#### PM distribution

Proteomic studies have shown that plant RBOHs are present in detergent-insoluble membrane fractions of the PM (Mongrand et al., 2004; Morel et al., 2006; Fujiwara et al., 2009; Stanislas et al., 2009). This suggests that they could be associated *in vivo* with sterol- and sphingolipid-rich PM microdomains also known as lipid rafts, like their animal counterparts (Jin et al., 2011). In line with this localization is the non-uniform distribution of several RBOHs within the PM of different cell types (Takeda et al., 2008; Liu et al., 2009; Lee et al., 2013; Noirot et al., 2014), and the discretely distributed RBOHD-dependent H_2_O_2_ patches observed along the PM of elicited tobacco cells (Lherminier et al., 2009).

Interestingly, RBOHD was recently found to form a complex with either of the PM immune receptors EFR or FLS2, and the receptor-like cytoplasmic kinase BIK1 (Kadota et al., 2014; Li et al., 2014). In response to MAMP elicitation, RBOHD is phosphorylated by BIK1, a post-translational modification required for its function in immunity against bacterial pathogens (Kadota et al., 2014; Li et al., 2014). It is tempting to speculate that the RHBOD-FLS2/EFR-BIK1 complex is part of a lipid raft-associated signaling platform since both RBOHD and FLS2 have already been found in detergent-insoluble membrane fractions (Mongrand et al., 2004; Keinath et al., 2010).

#### Subcellular dynamics

Reactive oxygen species species have also been detected in vesicles that migrate toward the site of infection. H_2_O_2_-containing vesicles accumulate at pathogen-challenge sites in the barley-powdery mildew interaction (Hückelhoven et al., 1999) and their incidence is influenced by mutations in the SNARE protein ROR2 (Collins et al., 2003; Figure 1). In tobacco cells, RBOHD has been found in two distinct intracellular compartments, one of them being the Golgi apparatus (Noirot et al., 2014). The Golgi-localized RBOHD pool is relocalized to the PM after stimulation with the oomycete elicitor cryptogein but prior to any transcriptional upregulation (Noirot et al., 2014). Internal reservoirs thus provide to the PM a supply of fresh enzymes to rapidly replace the PM-resident pool of RBOHD that is turned over following elicitor-induced activation, and to restore its signaling capacities. Recently, the PM dynamics of a GFP-tagged version of RBOHD were analyzed in *Arabidopsis* using variable-angle total internal reflection fluorescence microscopy (Hao et al., 2014). This study revealed that clathrin- and microdomain-dependent endocytic pathways cooperatively regulate the constitutive internalization of RBOHD. Furthermore, elicitation with flg22 was shown to increase the mobility and clustering of RBOHD, a potential prerequisite for its activation and subsequent internalization by endocytosis (Hao et al., 2014). The mechanism of endocytic turnover of RBOHD following cryptogein or flagellin elicitation remains, however, to be identified.

### SUGAR TRANSPORTERS

Obtaining metabolic resources such as sugars from their hosts is a key mechanism for symbiotic and pathogenic microorganisms to survive and reproduce. Access to nutrients depends upon microorganism’s lifestyle. For instance, mutualistic microbes or biotrophic pathogens will either grow in the apoplastic space and/or invade living cells of their hosts to get nutrients from the cytoplasm. There is some evidence suggesting that microbes manipulate the transport machinery of the host PM to increase the efflux of sugar and, that, in return, the host plant attempts to restrict the availability of apoplastic sugars to halt microbe proliferation and disease progression (Ruan, 2014).

The pioneering work of Chen et al. (2010b) has provided the first experimental evidence that bacterial pathogens manipulate the plant sugar transport system to fulfill its nutritional needs. Chen et al. (2010b) identified a new family of sugar uniporters called SWEETs that facilitate sugar efflux into the apoplast. They further showed that *Xanthomonas oryzae* pv. *oryzae* (*Xoo*) upregulates the expression of two SWEET genes in rice, a plant response that results from the direct binding of TAL (transcriptional activator-like) effectors to the SWEET promoters (Chen et al., 2010b). Since then, a third *SWEET* gene was shown to be induced after *Xoo* infection (Liu et al., 2011).

Silencing of the *N. benthamiana* squalene synthase gene, a key enzyme in the phytosterol biosynthesis pathway, results in compromised basal and non-host resistances, events that are correlated with an enhanced efflux of nutrients, mainly sugars, in the apoplastic space (Wang et al., 2012). In this study, it was suggested that alteration of specific PM sterols affects permeability and fluidity of the PM as previously demonstrated in artificial membranes (Schuler et al., 1991). One might imagine that the activity and/or abundance of sugar transporters at the PM are modified by the alteration of PM sterols and fluidity as in animal cells for transmembrane proteins (Bhor and Sivakami, 2003; Levitan et al., 2010), leading to bacterial proliferation.

Depriving microorganisms of the sugars they need by changing sugar fluxes toward host cells appears as a potential defense mechanism in the battle against pathogens. Active resorption of hexoses has been observed in elicited pine suspension cells (Azevedo et al., 2006). Expression of the monosaccharide H+ symporter STP4 is rapidly induced in elicited *Arabidopsis* suspension cells as well as in plants exposed to fungal pathogens (Truernit et al., 1996). Recently, the sugar transporter STP13 was shown to be induced in *Arabidopsis* leaves challenged with *Botrytis cinerea* (Lemonnier et al., 2014). Phenotypic analysis of a knockout mutant and plants constitutively-expressing STP13 revealed that this sugar transporter participates in host intracellular sugar uptake as a basal resistance mechanism against *B. cinerea* (Lemonnier et al., 2014).

Degradation of sugar efflux carriers after internalization by endocytosis is another potential mechanism to reduce apoplastic sugar concentration. Circumstantial evidence suggests that endocytic degradation of sugar carriers could operate in the defense against pathogens. On the one hand, targeting and cycling of the potato sucrose transporter 1 (SUT1) at the PM are controlled by endocytosis during plant development (Liesche et al., 2010; Krügel et al., 2012). On the other hand, glucose efflux is inhibited after cryptogein elicitation of tobacco cells (Bourque et al., 2002). The mechanism of inhibition is not known but as cryptogein stimulates endocytosis (Leborgne-Castel et al., 2008), it is tempting to speculate that such event may modify the abundance and/or the activity of glucose transporters in such a way that glucose uptake is inhibited.

In AM, the symbiotic fungus offers nutrients, such as phosphorus and nitrogen, to the plant and get, in return, carbohydrates produced by photosynthesis (Harrison, 1999). The tomato SUT2, which is expressed in colonized cells, ensures an essential function for the mycorrhizal symbiosis; the control of sucrose transport back to the plant cytosol to balance the needs of the host and its symbiont (Bitterlich et al., 2014). A recent search for SUT2-interacting proteins has revealed proteins either associated to detergent-insoluble membranes or localized in intracellular vesicles (Bitterlich et al., 2014). This result suggests that SUT2 may be recycled or degraded via a microdomain-associated endocytic pathway. A similar mechanism of internalization has been evidenced in a yeast expression system for the plant sucrose transporter SUT1 (Liesche et al., 2010). The detailed SUT2 targeting pathway to the PAM and its regulation, where active nutrient exchange occurs, remains to be fully investigated.

Interestingly, an RNA-seq transcriptomic approach has reported, in addition to host transporter genes such as SUTs and SWEET1, a highly significant upregulation of remorin genes in ectomycorrhizal interaction (Tarkka et al., 2013). It may suggest that modification of membrane properties by remorins could enhance PM targeting and/or activity of transporters for exporting sugars into the plant apoplast to support fungus growth.

### OPEN QUESTIONS

Interestingly, all PM proteins discussed above, and whose intracellular trafficking is modulated upon exposure to microorganisms, have been found in detergent-insoluble membranes or to co-localize with microdomain-associated proteins. Membrane microdomains are thought to temporally and spatially organize proteins and lipids into dynamic signaling complexes (Cacas et al., 2012). Such a functional link between PRR signaling and relocation into membrane microdomains has been suggested for the flagellin receptor FLS2 from proteomic studies (Keinath et al., 2010) but remains to be demonstrated *in vivo*. Alternatively, protein clustering in microdomains could promote internalization by endocytosis and thus extents signaling intracellularly or ensures its termination. Future studies will have to characterize the dynamics of association of proteins with microdomains at the PM but also in intracellular compartments to figure out the precise role of lateral compartmentalization of membranes in pathogenic or mutualistic interactions.

As endosomal signaling is now an established process in the defense reactions against pathogens, another open question is whether signaling endosomes are genuine targets for effectors produced by adapted pathogens. Recently, the HopW1 effector has been shown to increase the growth of *Pseudomonas syringae* on *Arabidopsis* plants through the disruption of the actin cytoskeleton and the inhibition of endocytosis (Kang et al., 2014). We may hypothesize that disruption of actin-dependent endocytosis reduces endosomal signaling (i.e., from PRRs) and thus leads to plant infection. Identification of the intracellular targets of microbial secretory effectomes might shed some lights on this issue.

## CONCLUSION

This review highlights the dynamics of the PM as a key process for a better molecular dialog between the plant cell and its biotic environment.

Intracellular colonizers (mutualists or pathogens) trigger a massive remodeling of the host cell PM that leads to the formation, and maintenance, of a perimicrobial membrane with a unique identity. Perimicrobial membranes are characterized by the presence of microdomain-associated proteins of the remorin and flotillin families. These proteins are believed to play a role as molecular scaffolds for the building of signaling platforms. Future should reveal whether they are involved in the polarized delivery of specific PM proteins and/or the endocytic retrieval of others.

Recent studies have identified vesicle trafficking regulators as modulators of the plant response to pathogenic or mutualistic microorganisms. A challenge that lies ahead will be to identify commonalities and differences in the vesicle pathways implicated in the different types of interactions. It will also be important to determine to what extent pathogen effectors affect the accumulation of PM proteins for subversion of plant defense. Deciphering new mechanisms of pathogenicity as well as novel aspects of plant immunity would benefit from the identification of the whole repertoire of protein trafficking components that are targeted by pathogen effectors. The identification of new R-genes should help to design novel strategies for manipulating crop plants toward resistance to ravaging pathogens.

### Conflict of Interest Statement

The Guest Associate Editor Daniel Wipf declares that, despite being affiliated to the same institution as the authors, the review process was handled objectively and no conflict of interest exists. The authors declare that the research was conducted in the absence of any commercial or financial relationships that could be construed as a potential conflict of interest.

## Abbreviations

AM: arbuscular mycorrhiza
ARF: ADP-ribosylation factor
EFR: elongation factor-TU receptor
EHM: extrahaustorial membrane
ER: endoplasmic reticulum
ETI: effector-triggered immunity
flg22: 22-amino acid sequence of the flagellin
FLS2: FLAGELIN SENSING 2 receptor
IT: infection thread
MAMP: microbe-associated molecular pattern
MVB: multivesicular bodies
PAM: periarbuscular membrane
PAMP: pathogen-associated molecular pattern
PBM: peribacteroid membrane
PM: plasma membrane
PRR: pattern-recognition receptor
PTI: PAMP-triggered immunity
RAC: Ras-related C3 botulinum toxin substrate
RBOH: respiratory burst oxidative homolog
ROS: reactive oxygen species
SA: salicylic acid
SNARE: *N*-ethylmaleimide-sensitive factor attachment protein receptor
TGN: *trans*-Golgi network.
